# A flexible tool to plot a genomic map for single nucleotide polymorphisms

**DOI:** 10.1186/s13029-016-0052-z

**Published:** 2016-04-02

**Authors:** Fuquan Zhang

**Affiliations:** Wuxi Mental Health Center, Nanjing Medical University, 156 Qianrong Road, Wuxi, Jiangsu Province 214151 China

**Keywords:** R package, Mapsnp, SNP map

## Abstract

**Background:**

Most genetic association studies use single-nucleotide polymorphisms (SNPs) as the research targets. However, resources to visualize the genomic map of candidate SNPs in a programming manner are limited. We have previously created an R package, mapsnp v0.1, to plot the genomic map for a panel of SNPs within a genomic region of interest. It failed to work under the latest version of Gviz package.

**Results:**

We updated the mapsnp package to keep up with the latest package environment and improved its functionality by adding more parameters to fine tune plotting outputs.

**Conclusions:**

The mapsnp package is a flexible software to visualize genomic map for SNPs, involving the relative chromosome location and the transcripts in the region.

**Electronic supplementary material:**

The online version of this article (doi:10.1186/s13029-016-0052-z) contains supplementary material, which is available to authorized users.

## Background

Single-nucleotide polymorphisms (SNPs) are the most common type of genetic variation among people. SNPs are used for estimating predisposition to disease.

Visualizing genomic map relevant to SNPs may inform the reader intuitively. Genome browsers are common tools to show a SNP’s genomic information, including NCBI genome browsers [1], UCSC [2], and Ensembl Genome Browser [3]. These browsers offer retrieval resources and serve as reference datasets for individual SNPs or genes. UCSC and Ensembl Browser offers a set of annotation ‘tracks’ for a genomic region. However, they are not programmatically accessible and have limited plotting options to render users’ data. With these tools, it is not possible to produce a map for a specific set of SNPs.

The R language [4] is a widely used language and software environment for statistical computing and graphics. Several programming tools have been developed under R environment for visualizing genomic data, including GenomeGraphs [5], ggbio [6], and Gviz [7]. Within these packages, individual types of genomic features or data are represented by separate tracks, and there are constructor functions to coordinate and plot these tracks. However, none of these packages provide a method specified to plot genomic information for a panel of user-supplied SNPs.

To fulfill this need, we have developed mapsnp v0.1 [8] to plot genomic maps for SNPs. It works under R v2.15 and Gviz v1.2.1. As the upgrading of R, the Gviz package was also updated, which deprecated our mapsnp package. To keep up with the latest R environment and the Gviz package, we created mapsnp v0.2.

## Implementation

The mapsnp package leverages the Gviz system [7] to plot a genomic map for SNPs. A SNP map includes five tracks, an ideogram track for a chromosome, an axis track for genomic coordinates, a transcript track for relevant transcripts, a SNP location track, and a SNP label track annotating their ID symbol.

The mapsnp v0.2 package contains one function, ‘msb’. The function has three mandate parameters, ‘M’, ‘start’, and ‘end’. Parameter ‘M’ is a data frame consist of three columns, including chromosome, SNP ID, and SNP genomic location. The ‘start’ and ‘end’ parameter define the range of a highlighting region, typically a gene region where the SNPs are located to. For transcript track, the ‘msb’ function utilizes Homo Sapiens data from UCSC build hg19 based on the knownGene table, implemented in the ‘TxDb.Hsapiens.UCSC.hg19.knownGene’ package [9].

There are dozens of other parameters, which fine-tune other track properties, such as color, size, track name, annotation text, and so on. The detailed usage of the package is described in Additional file 1.

## Results and discussion

**Fig. 1:**
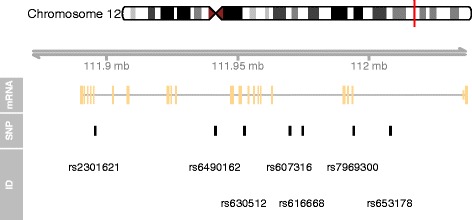
A concise genomic map for seven SNPs within ATXN2 using UCSC database. At the top, the relevant chromosome is drawn with the subregion of interest marked in red. The ‘mRNA’ track shows the combined gene model of the alternative transcripts of the ATXN2 gene. At the bottom, the SNPs’ location and ID are plotted along the same genomic coordinate

To illustrate the use of mapsnp v0.2, we show an example for ‘msb’ on the built-in dataset involving seven candidate SNPs within the ATXN2 gene (Fig. 1) [10]. The genomic range of this gene is from 111950277 to 112036294 base pair.

> library (mapsnp)

> library (TxDb.Hsapiens.UCSC.hg19.knownGene)

> data (snp)

> msb (M = snp, start = 111950277, end = 112036294)

Compared with its precursor, mapsnp v0.2 offers more parameters to fine-tune the output map. See Additional file 2 for several examples using alternative plotting options.

SNPs occur normally throughout a person’s genome. They can act as biological markers, helping scientists locate genes that are associated with disease. Visualization of SNPs facilitates exploration and discovery by revealing genomic patterns of variations [6]. mapsnp provides an easy-to-use method to visualize genomics annotations for a group of SNPs. The output maps deliver views of SNP locations, genomic regions, summary views of splicing patterns, and genome-wide overviews with karyogram. The package is especially useful for most candidate gene studies by exploring genomic features for relevant SNPs.

Widely-used visualization tools are implemented in the form of a genome browser. Comparison of our tool with UCSC Genome Browser and Ensembl has been described previously [11]. Of note is that the package can handle only one chromosome at a time. Also, users need an established internet connection to fetch data from UCSC.

## Conclusion

The mapsnp package provides a simple and flexible function to plot genomic maps for a set of SNPs.

## Availability and requirements

Project name: mapsnp

Project home page: https://sourceforge.net/projects/mapsnp/files/?

Operating system(s): Platform independent.

Programming language: R platform.

Other requirements: Internet connection.

License: GPL (≥3)

## Additional files

Additional file 1: mapsnp manual. (PDF 90 kb) Additional file 2: Plotting examples. Examples to plot the map with other options. (PDF 193 kb)
