# Supplementary material for: A flexible tool to plot a genomic map for single nucleotide polymorphisms
Source: Source Code Biol Med. 2016 Apr 2;11:5. doi: 10.1186/s13029-016-0052-z (PMC4818925; doi:10.1186/s13029-016-0052-z)
Supplement: Additional file 1: — mapsnp manual. (PDF 90 kb) [file 13029_2016_52_MOESM1_ESM.pdf]

Package ‘mapsnp’

January 17, 2015

Type Package

Title Plot a Genomic Map for Single Nucleotide Polymorphisms

Author Fuquan Zhang [aut, cre]

Maintainer Fuquan Zhang <fq.z@qq.com>

Depends R (>= 3.1.0)

Imports Gviz

Suggests TxDb.Hsapiens.UCSC.hg19.knownGene

Description A package to plot a genomic map of single nucleotide polymorphisms (SNPs), including the a chromosome ideogram, transcripts of the gene on which the SNPs map to, the genomic location of SNPs, and their ID number.

License GPL (>= 3)

Encoding UTF-8

LazyLoad yes

Version 0.2

Date 2015-01-17

R topics documented:

mapsnp-package 1
msb 2
snp 5

Index 6

`mapsnp-package` *The mapsnp Package*

Description

Plot a Genomic Map for Single Nucleotide Polymorphisms.

Details

Package:	mapsnp
Type:	Package
Version:	0.2
Date:	2015-01-17
Depends:	R (>= 3.1.0)
Encoding:	UTF-8
License:	GPL (>= 3)
LazyLoad:	yes

A package to plot a genomic map of single nucleotide polymorphisms (SNPs), including the a chromosome ideogram, transcripts of the gene on which the SNPs map to, the genomic location of SNPs, and their ID number.

Author(s)

Fuquan Zhang

`msb` *Plot a genomic map for snp*

Description

A function to plot genomic map of snp using dataset from R package "TxDb.Hsapiens.UCSC.hg19.knownGene". The trancript track includes only major trancripts within a highlighting range.

Usage

```
msb(M, start = start, end = end, extend.left = 0, extend.right = 0,
  cex = 0.6, showId.chr = TRUE, showBandId = TRUE, fontsize.chr = 12,
  fontcolor.chr = "black", labelPos.axis = "below", littleTicks = FALSE,
  geneName = "mRNA", showLab.gene = FALSE, stackHt.gene = 0.5,
  fill.gene = "#FFD58A", col.line = "darkgray", min.width = 1,
  lwd.gene = 1, snpName = "SNP", snpLab = 1:nr, showLab.snp = FALSE,
  snpWd = 1, fill.snp = "black", stackHt.snp = 0.4, IDName = "ID",
  showLab.ID = TRUE, IDWd = 1, IDPos = "below", stackHt.ID = 0.2,
  fontcolor.item = "black", col.ID = "transparent", rotation.item = 0,
  stacking = "dense", genome = "hg19", reverseStrand = FALSE,
  fontcolor.title = "white", fontsize.title = 12,
  background.title = "grey", ...)
```

Arguments

`M`	A three-column matrix or data.fram. The first column is Chromosome, e.g. 1, 2, 3, ..., X, Y, the second column is snp ID, and the third column is snp genomic location.
`start`	An integer scalar with the genomic start coordinates for the highlighting range.
`end`	An integer scalar with the genomic end coordinates for the highlighting range.

`extend.left`	Numeric scalar, extending the plotting range to the left. In addition to positive or negative absolute integer values one can also provide a float value between -1 and 1 which will be interpreted as a zoom factor, i.e., a value of 0.5 will cause zooming in to half the currently displayed range.
`extend.right`	Numeric scalar, extending the plotting range to the right. In addition to positive or negative absolute integer values one can also provide a float value between -1 and 1 which will be interpreted as a zoom factor, i.e., a value of 0.5 will cause zooming in to right the currently displayed range.
`cex`	Numeric scalar. The overall font expansion factor for the axis annotation text. Default to 0.6.
`showId.chr`	Logical scalar. Control whether to plot the chromosome ideogram track item identifiers. Default to TRUE.
`showBandId`	Logical scalar. Show the identifier for the chromosome bands if there is space for it. Default to FALSE.
`fontsize.chr`	Numeric scalar. The font size for the chromosome name text. Default to 16.
`fontcolor.chr`	Character scalar. The font color for the chromosome name text. Default to 'black'.
`labelPos.axis`	Character vector, one in "alternating", "above" or "below". The vertical positioning of the axis labels. Default to "below".
`littleTicks`	littleTicks=FALSE: Logical scalar. Add more fine-grained tick marks. Default to FALSE.
`geneName`	Character scalar of the transcript track name used in the title panel when plotting. Default to "Transcripts".
`showLab.gene`	Logical scalar. Control whether to plot the transcripts track item identifiers. Default to FALSE.
`stackHt.gene`	Numeric between 0 and 1. Controls the vertical size and spacing between stacked elements. The number defines the proportion of the total available space for the stack that is used to draw the transcripts items. Default to 0.5.
`fill.gene`	Character or integer scalar. The fill color for untyped items. This is also used to connect grouped items.
`col.line`	Character or integer scalar. The color used for gene track line elements.
`min.width`	Numeric scalar. The minimum range width in pixels to display. All ranges are expanded to this size in order to avoid rendering issues.
`lwd.gene`	Integer scalar. The line width for gene track items. This is also used to connect grouped items.
`snpName`	Character scalar of the snp location track name used in the title panel when plotting. Default to "snp".
`snpLab`	item labels for snp location. Default to series from 1 to number of rows.
`showLab.snp`	Logical scalar. Control whether to plot the snp location track item identifiers. Default to FALSE.
`snpWd`	Integer vectors, times of width relative the 1/140 of length of the genetic region for the snp location track items. Default to 1.
`fill.snp`	Character or integer scalar. The fill color for snp location items. Default to "black".
`stackHt.snp`	Numeric between 0 and 1. Controls the vertical size and spacing between stacked elements. The number defines the proportion of the total available space for the stack that is used to draw the snp location items. Default to 0.4.

IDName	Character scalar of the snp ID track name used in the title panel when plotting. Default to "ID".
showLab.ID	Logical scalar. Control whether to plot the snp ID track item identifiers. Default to TRUE.
IDWd	Integer vectors, times of width relative the 1/20 of length of the genetic region for the snp ID track items. Default to 1.
IDPos	Character vector, one in "alternating", "above" or "below". The vertical positioning of the snp ID labels. Default to "below".
stackHt.ID	Numeric between 0 and 1. Controls the vertical size and spacing between stacked elements. The number defines the proportion of the total available space for the stack that is used to draw the snp ID items. Default to 0.2.
fontcolor.item	Character or integer scalar. The font color for item identifiers.
col.ID	Character or integer scalar. The border color for all track items.
rotation.item	Numeric scalar. The degree of text rotation for item identifiers.
stacking	Object of class "character", the stacking type of overlapping items on the final plot. One in c(hide, dense, squish, pack,full). Currently, only hide (do not show the track items at all), squish (make best use of the available space) and dense (no stacking at all) are implemented. Default to "dense".
genome	The genome on which the track ranges are defined. Usually this is a valid UCSC genome identifier. Default to "hg19".
reverseStrand	By default all tracks will be plotted in a 5' -> 3' direction. It sometimes can be useful to actually show the data relative to the opposite strand. To this end one can use the reverseStrand display parameter, which does just what its name suggests. Default to FALSE.
fontcolor.title	A character, background color of track title. Default to "white".
fontsize.title	Numeric scalar. The font size for track titles. Default to 16.
background.title	A character, background color of track name. Default to "grey".
...	Additional items which will all be interpreted as further display parameters. See settings and the "Display Parameters" section below for details.

Details

A function to plot genomic map of snp using dataset of UCSC.hg19

Examples

```
library(Gviz)
library(TxDb.Hsapiens.UCSC.hg19.knownGene)
data(snp)
msb(M=snp, start=111950277, end=112036294)
```

snp *An example dataset*

Description

SNP map information including chromosome, SNP ID, and SNP location.

Usage

```
data(snp)
```

Format

A data.frame containing genomic information for 7 SNPs in the ATXN2 gene.

Source

Association analysis of a functional variant in ATXN2 with schizophrenia. Neurosci Lett. 2014.

References

Zhang F, et al. Association analysis of a functional variant in ATXN2 with schizophrenia. Neurosci Lett. 2014; 562: 24-7.

Index

∗Topic **datasets**
    snp, 5
∗Topic **package**
    mapsnp-package, 1

mapsnp-package, 1
msb, 2

snp, 5
