# Supplementary material for: A flexible tool to plot a genomic map for single nucleotide polymorphisms
Source: Source Code Biol Med. 2016 Apr 2;11:5. doi: 10.1186/s13029-016-0052-z (PMC4818925; doi:10.1186/s13029-016-0052-z)

Examples to adjust the output map

The following examples shows some options to tune the final map.

Change the ID position into "alternating".

> msb (M = atxn2, start = 111950277, end = 112036294, IDPos='alternating')

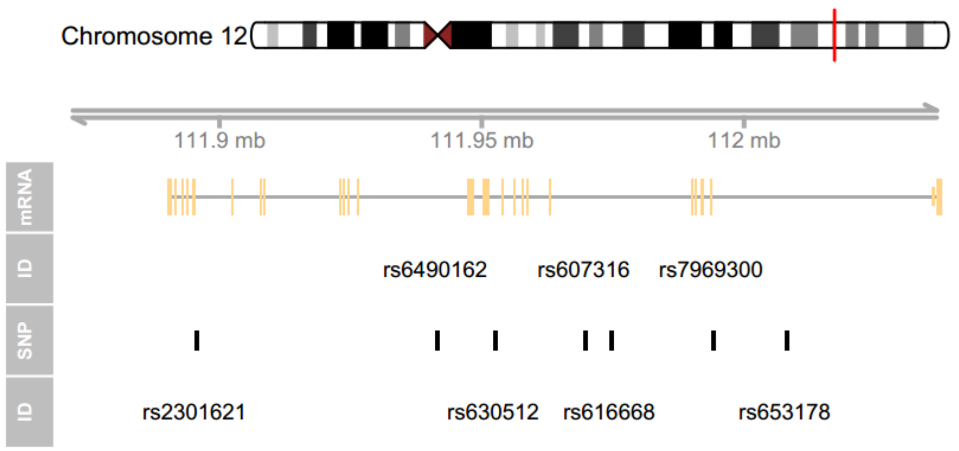

Rotate the direction of SNP ID label into vertical.

> msb (M = atxn2, start = 111950277, end = 112036294, rotation.item = 90, IDWd = 0.1)

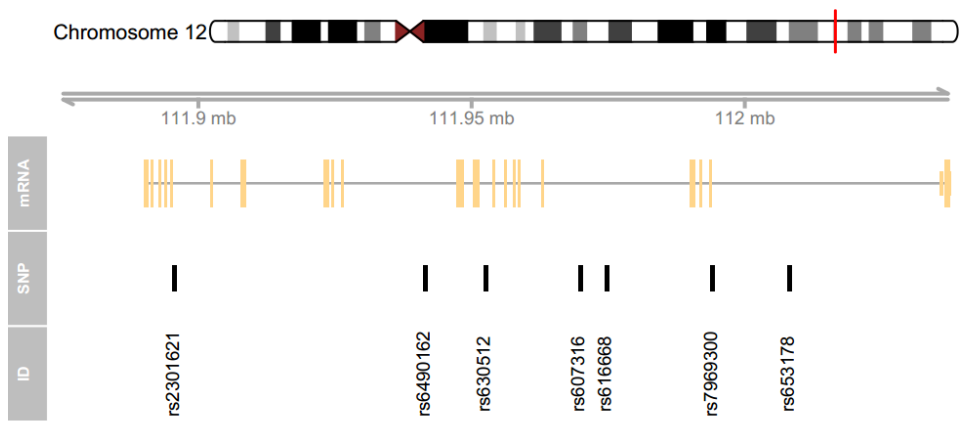

Change background color and background color of track title.

> msb (M = atxn2, start = 111950277, end = 112036294, rotation.item = 90, IDWd = 0.1, fontcolor.title = 'yellow', background.title = "lightblue")

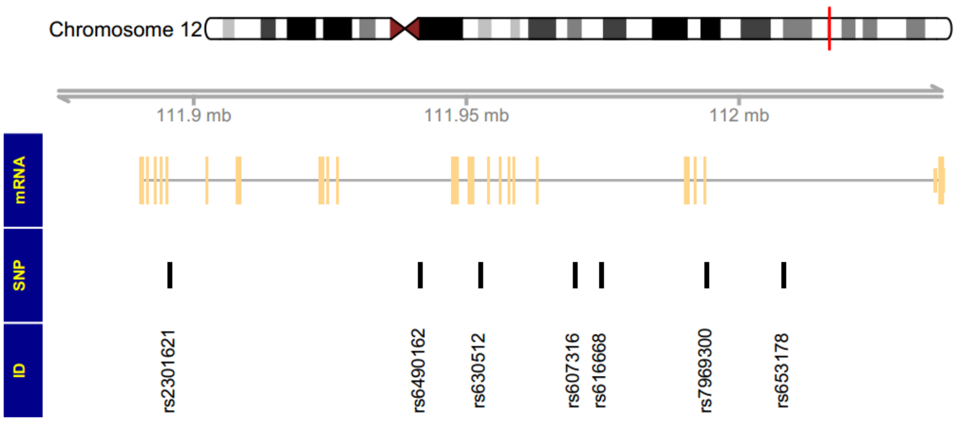

Change background color of track title into "brown" and background color of track title into "white".

> msb (M = atxn2, start = 111950277, end = 112036294, rotation.item = 90, IDWd = 0.1, fontcolor.title = 'brown', background.title = "white")

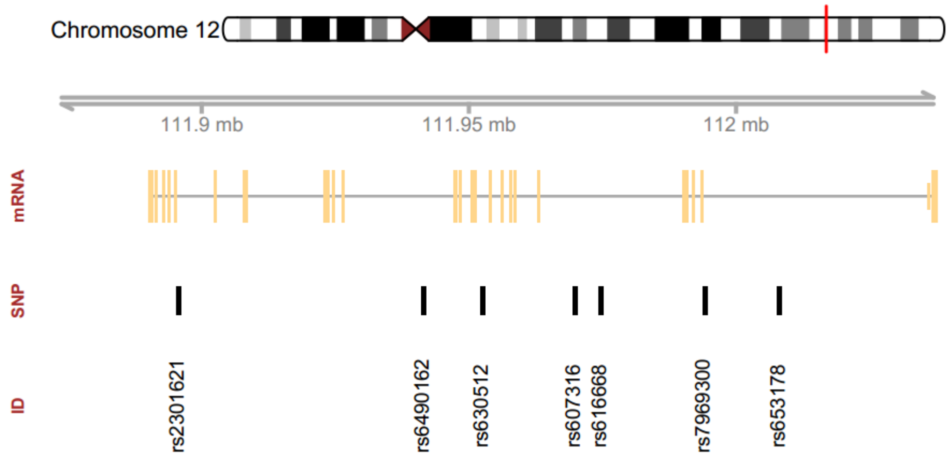

Change the gene track name into "ATXN2".

> msb (M = atxn2, start = 111950277, end = 112036294, rotation.item = 90, IDWd = 0.1, geneName = 'ATXN2')

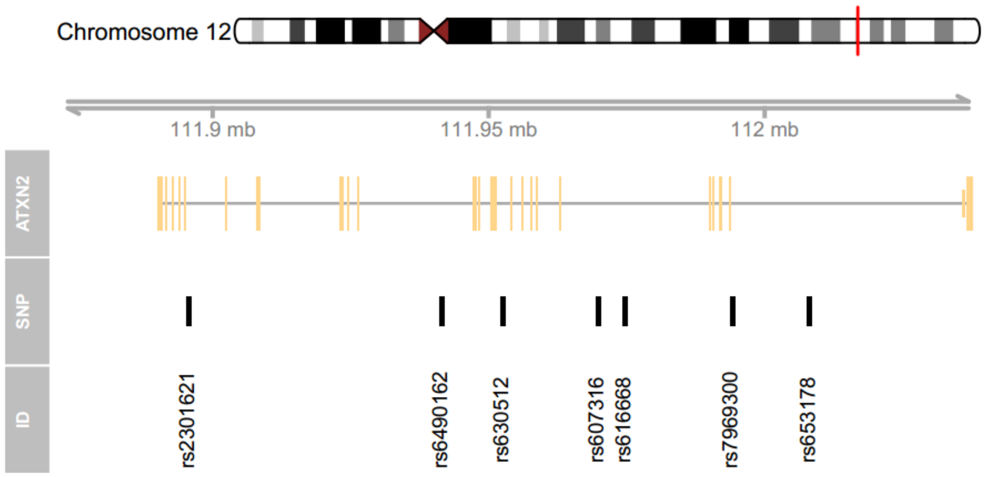

Highlight the sixth SNP using red color.

> color.snp = rep ('black', 7); color.snp [6] = 'red'

> msb (M = atxn2, start = 111950277, end = 112036294, rotation.item = 90, IDWd = 0.1, fill.snp = color.snp)

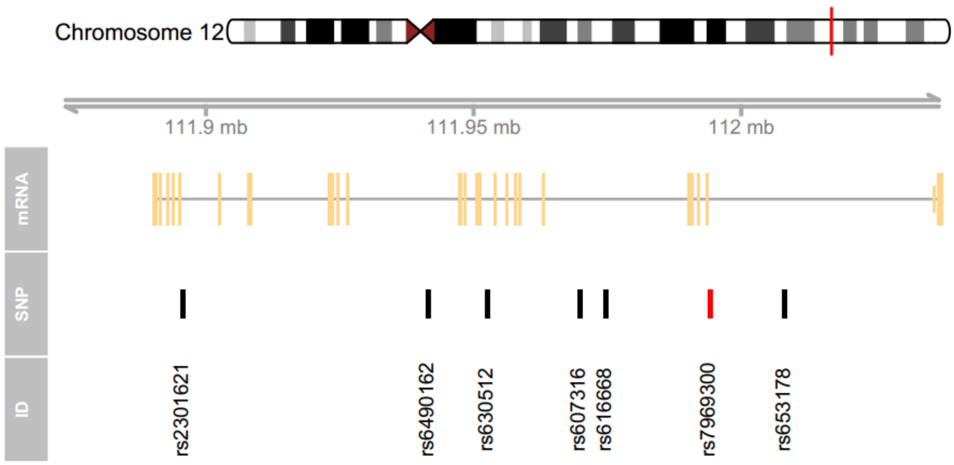

Add more fine-grained tick marks, and change the positioning of the axis labels into "alternating".

> msb (M = atxn2, start = 111950277, end = 112036294, rotation.item = 90, IDWd = 0.1, littleTicks = TRUE, labelPos.axis = "alternating")

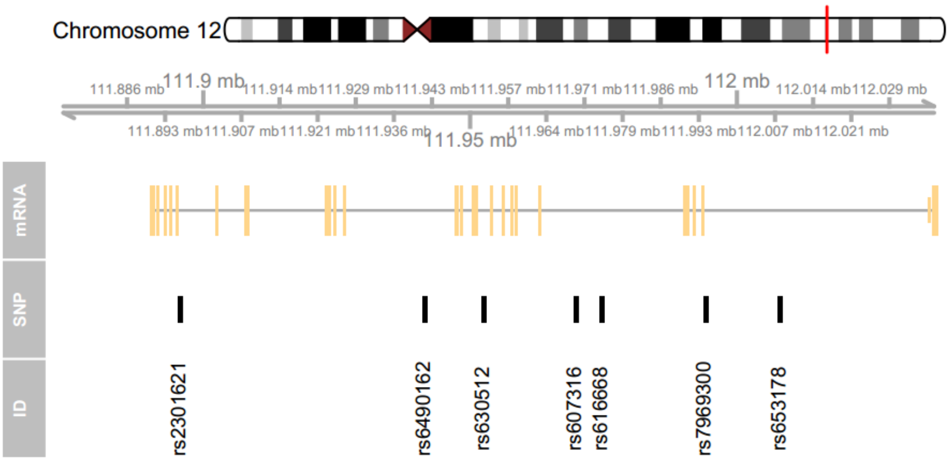

Supplement: Additional file 2: — Plotting examples. Examples to plot the map with other options. (PDF 193 kb) [file 13029_2016_52_MOESM2_ESM.pdf]
